# Health Impact of Heavy Metals in Samples of Dried Fruits in Iraq

**DOI:** 10.1155/ianc/3934929

**Published:** 2025-12-07

**Authors:** Qunoot Mohammed Hadi, Faten A. Mahdi, Farah J. Hamood, Saif Hamid Abbas, Ali Abid Abojassim

**Affiliations:** ^1^ Department of Physics, College of Education for Pure Sciences, University of Babylon, Babylon, Iraq, uobabylon.edu.iq; ^2^ Faculty of Physics, Doctoral School on Physics, University of Bucharest, Bucharest, Romania, unibuc.ro; ^3^ Department of Quality Assurance, University of Babylon, Hillah, Iraq, uobabylon.edu.iq; ^4^ Department of Physics, Faculty of Science, University of Kufa, Kufa, Iraq, uokufa.edu.iq

**Keywords:** Iraqi markets, cancer risk, Cd, Cr, dried fruit, Pb

## Abstract

This study was carried out to assess the health hazards associated with the amount of the heavy metal lead (Pb), which is one of them, and samples of chromium (Cr) and cadmium (Cd) of dried fruits sold in Iraqi marketplaces. We gathered and used atomic absorption spectrometry (AAS) to assess 15 different samples of dried fruits that were imported from Iran. The average metal concentrations were as follows: (Pb = 0.6416 mg/kg), (Cd = 0.1910 mg/kg), and (Cr = 0.3544 mg/kg), according to the data. A number of samples were found to be above the FAO/WHO maximum allowable levels, which are 0.12 mg/kg for chromium and 0.05 mg/kg for cadmium. Apple (Cd = 0.897 mg/kg), peach (Cr = 1.289 mg/kg), and quince (Pb = 1.951 mg/kg) had the greatest values. Pb, Cd, and Cr had average estimated daily intakes (EDIs) of 0.4584, 0.1502, and 0.2532 mg/kg/day, respectively, according to the health hazard indices that were also constructed. Cd = 0.1364, Cr = 0.084, and Pb = 0.131 were the average target hazard quotient (THQ) index. The average hazard index (HI) for all metals was less than 1, at 0.351, showing no direct noncarcinogenic health harm associated with regular use. The possibility of long‐term cumulative hazards, however, is indicated by samples that exceed the allowable levels, necessitating stricter food control of imported goods. The TCR (carcinogenic risk) values for lead (Pb), chromium (Cr), and cadmium (Cd) levels were assessed in dried fruit samples. The average overall TCR value was 5.106 ± 1.10, with a range of 0.002 × 10^−6^ to 16.90 × 10^−6^. Every one of these values falls below the acceptable ranges (10^−6^ and 10^−4^) established by the US Environmental Protection Agency (USEPA), suggesting that eating these fruits did not significantly increase a person’s chance of developing cancer. This is one of the rare studies to focus on dried fruits in Iraq, despite the fact that there have been earlier international studies on heavy metals in fruits. In accordance with the international standards, such as THQ, HI, and TCR, it integrates chemical assessment with health analysis to produce reliable data that back up the nation’s food control initiatives.

## 1. Introduction

Consuming the utmost quantity of vitamins, dietary fiber, and nutrients is crucial, and fruits are an important part of any diet. [1]. There is a need to evaluate the quality and safety of dried fruits, especially in relation to metal contamination, given the growing demand for them as a long‐term substitute for peeled fruits. Because they are good for human health, they are regarded as a part of fruits and plants. The functional and physicochemical characteristics of fruit components, which support these health advantages, can be greatly impacted by extraction and processing techniques. [2]. Global scientific research shows that industrial pollution has increased chromium (Cr), cadmium (Cd), and lead (Pb) concentrations, among other dangerous metals, in fruits and fruit products, unsound farming practices, and the use of tainted irrigation water [3]. This work is noteworthy as it is the first in Iraq to crystallize the metal levels in a collection of dried fruits. It employs a thorough analytical method that thoroughly examines every component. It contributes to one‐sixth of the accurate and reliable knowledge of food safety in Iraq and provides a preliminary database that can be built upon in further studies or in a more rigorously controlled environment [2]. Certain trace elements may be hazardous based on their concentration. Their half‐life is long, and they can accumulate in biological networks. Due to their abundance in soil, water, sediments, and the environment, in food chains, Cd, Cr, Ni, and Pb accumulate and enter the human body. Numerous sources of industrial and urban waste can release these metals into groundwater and agricultural land, including the improper handling of building waste, a major known source of environmental heavy metals in developing nations [4]. These metals have been identified in agricultural goods at worrying amounts in a number of studies conducted in nations, including China, India, Turkey, and Iran, purposefully hiding any possible health hazards, particularly when consuming food [5–8]. Given their concentration, certain trace elements could be dangerous [9]. Because of their long half‐life, they can accumulate in biological networks. Lead, nickel, chromium, and cadmium build up in food chains and get inside people because of their prevalence in soil, water, sediments, and the environment. Cadmium has detrimental effects, such as oxidative stress with an increased chance of developing cancer [10]. Chromium could cause irritation to epithelial cells [11]. Nickel, for instance, can cause cardiac issues and lung cancer [12]. Certain trace elements can build up in biological networks and have a lengthy half‐life, making them dangerous depending on their quantity. These metals can enter the body through food and end up in tissues for storage. One of the most dangerous of these metals is cadmium because it causes oxidative stress. Its harmful effects on the kidneys and bones are accompanied by an innate immune system activation with an increased chance of developing cancer [13, 14]. This substance causes cancer, the International Agency for Research on Cancer [15]. Investigating the negative and positive impacts of metal consumption on human health is necessary. To determine food safety risk levels, the number of hazardous metals in the consumed dried fruit must be taken into account. Thus, the study’s objectives were to determine the concentrations of lead, cadmium, and chromium in dried fruits in Iraq and evaluate the health hazards related to eating them by figuring out dietary exposure levels. We compared the outcomes for the target hazard quotient (THQ), hazard index (HI), and total cancer risk (TCR) to calculate the estimated daily intake (EDI). Moreover, the data were assessed after statistical analysis. Based on the above, there is an urgent need to conduct field studies to evaluate the heavy metal concentrations in dry foods available in local markets and determine their association with potential health risks.

## 2. Materials and Procedures

### 2.1. Sample of Collection

**Figure 1:**
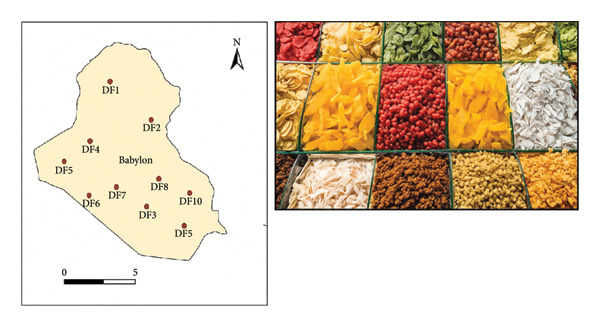
Fifteen samples of dried fruits from Iraqi markets and location.

Fifteen different illustrations of dried fruits (peach, fig, coconut, grape, orange, nectarine, pear, watermelon, plum, berry, apple, kiwi, banana, pomegranate, and quince) were collected from the local Iraqi markets during February 2025 as shown in Figure 1. From Figure 1, it is shown that 15 samples of dried fruit, coded from DF1 to DF15, were situated in a map of Iraq. These samples represent various brands commonly available in the markets and were selected using a nonprobability convenience sampling method to ensure representation of the most commonly consumed types. Each sample represents a single 500‐g product. No replicates were used at this stage; each sample was analyzed as an individual sample representing an independent unit in the market.

### 2.2. Sampling Preparation

To obtain a constant weight, they were dried for 20 min at 70°C in an electric oven, and the samples were first cleansed with distilled water to get rid of dust and contaminants.

**Figure 2:**
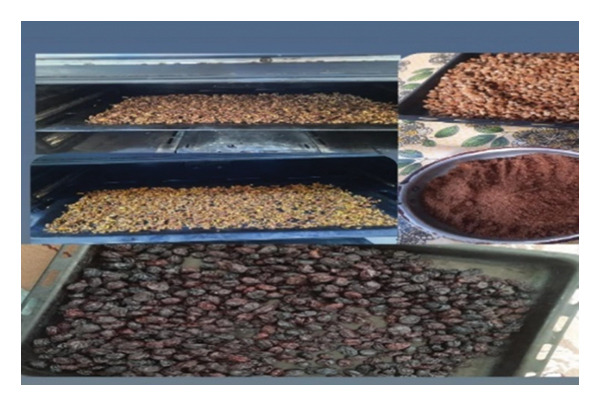
Sampling preparation.

Upon drying as shown in Figure 2, the samples were ground using an electric grinder and then sieved to obtain a homogeneous powder. The process of digestion was completed. After combining nitric acid (HNO_3_) and perchloric acid (HClO_4_) in a 3:1 ratio, 1 g of the mixture was weighed and placed in a glass container. After adding 10 mL of the acid combination, the mixture was heated steadily until the solution became transparent. After cooling, the fluid underwent filtration and dilution with mineral‐free distilled water to make 25 mL.

### 2.3. Absorption Spectrometer for Atoms

**Figure 3:**
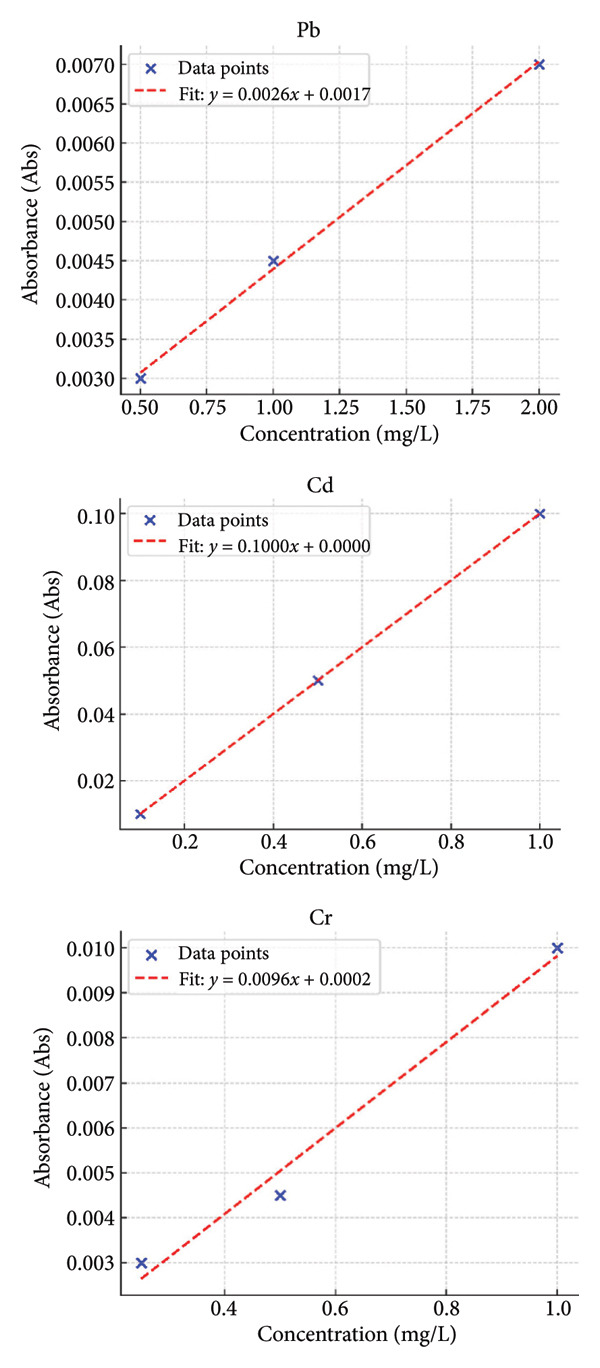
The heavy metal calibration curve used in this investigation.

A Shimadzu AA‐7000 atomic absorption spectrophotometer (AAS) was used to quantify the amounts of Pb, Cd, and Cr in the samples. Standard solutions from a reputable business (such as Merck) with known concentrations were used for calibration. Blank analysis was performed for each batch of samples. To confirm the analysis’s accuracy, certified reference materials (CRMs) were used. The samples were subjected to recovery testing using internal standards, and recovery rates varied from 92% to 98%. To guarantee repeatability and minimize error, each sample was examined three times. Detection limits (LOD) and quantification limits (LOQ) were as follows: for lead: LOD = 0.03–0.1 ppm; LOQ range = 0.1–0.4 ppm; for Cd: LOD = 0.002–0.008 ppm; LOQ = 0.007–0.03 ppm; and for Cr: LOD = 0.005–0.02 ppm; LOQ = 0.02–0.07 ppm. As advised by the IUPAC, these values were calculated statistically with the LOD standard deviation three times and the blank standard deviation 10 times [16]. The calibration curve for the standard for each element is displayed in Figures 3(a), 3(b), 3(c).

### 2.4. Health Risk Parameters

Many healthy risk indicators, such as the HI, EDI, and THQ, were determined due to the fact that samples of dried fruit that included heavy metals, including Pb, Cd, and Cr, are as follows:

EDI resulting from heavy metals in dry fruit samples calculated using equation (1), which is dependent on the weight of the human body (*B* *W*), the proportion of heavy metals in dried fruit (*C*_metal_), and the average daily dietary intake (*W*), is as follows [17, 18]:

(1) EDImgkgper day=Cmetalmg/kg×Wkg/dayBW kg,

BW value employed in this investigation was 0.05 kg/day for an adult whose average weight was 70 kg [19–21].

According to the Environmental Protection Agency (EPA), based on the oral reference dosage (RfD) and EDI, the THQ levels in the samples were calculated using the following formula [22, 23]:

(2) THQ=EDI mg/kg per dayRfDmg/kg per day.

For Cr, Cd, and Pd, the RFD values expressed in mg/kg daily were 3.5 × 10^−3^, 1 × 10^−3^, and 3 × 10^−3^, respectively [3, 24].

The values of HI brought on by all heavy metals can be found using the total THQ in this investigation using the following equation [25]:

(3) HImgkgper day=∑THQ.

According to the USEPA, the population’s CR from heavy metal exposure was computed using the formula below, which depends on a number of factors, for example, exposure duration (ED), exposure frequency (EFr), EDI (an acronym), average time (AT), and oral carcinogenic slope factor (CSFo), as explained in Ref. [26]:

(4) CR=EFrdays/year×EDyear×EDImg/kg per day×CSFomg/kg per dayATday/year×70 year×10−3.

The total cancer risk (TCR) values in this study can be calculated using the total of the CR caused by all heavy metals using the following equation [26]:

(5) TCR=∑CR.

Within this investigation, the values for EFr, ED, and AT were 350 days annually, 30 years, and 365 days annually × 70 years, respectively [27]. However, for Pb, Cd, and Cr, the CSFo value in units of mg/kg per day was 0.0085, 15, and 41, respectively [28].

### 2.5. Analysis of Statistics

The statistical analysis was performed using the SPSS statistical software package (SPSS Inc., Chicago, Illinois, USA) for Windows Version 20.

## 3. Results and Discussion

**Figure 4:**
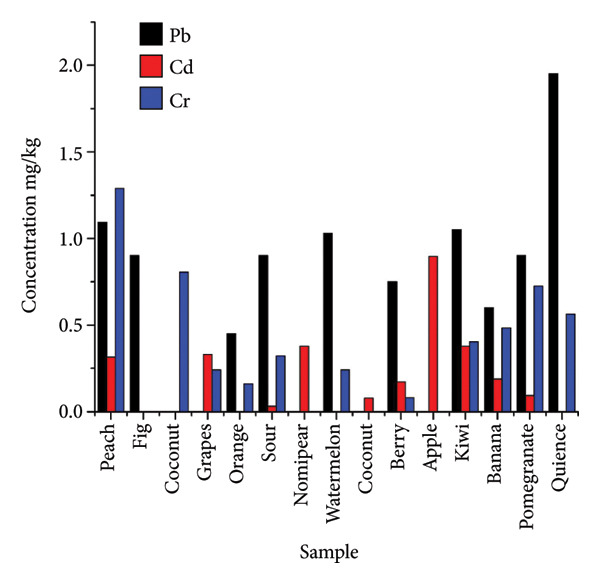
Averages of Pb, Cd, and Cr levels in samples.

AAS methods were used to detect heavy metals, such as Pb, Cd, and Cr in a number of dried fruit samples that were made in Iran and offered for sale in Iraqi markets. Table 1 and Figure 4 display the Pb, Cd, and Cr contents for 15 distinct Iranian‐made dry fruit samples that were gathered from Iraqi marketplaces. The levels of lead (Pb), Cd, and Cr in a number of dried fruit samples are listed in Table 1 and were discovered in many samples at levels between ND (no detection) and 1.951 mg/kg, averaging 0.6416 mg/kg. Every value is within the lower limit (10 mg/kg); however, it can be a sign of moderate contamination. The value in sample (DF15 [quince] = 1.951 mg/kg) was the highest. Cadmium (Cd) samples with concentrations between ND and 0.897 mg/kg and an average of 0.1910 mg/kg were discovered in significant amounts that were above the limit, reaching 0.05 mg/kg, for example, peach ([DF1] = 0.315), apple ([DF11] = 0.897), and banana ([DF13] = 0.378). This suggests a hazardous degree of cadmium pollution, as even at low amounts, this metal is extremely poisonous. Chromium (Cr) has an average of 0.3544 mg/kg and ranges from ND (no detection) to 1.289 mg/kg. An allowable limit of 0.12 mg/kg was clearly exceeded in a number of samples (DF1 [peach] = 1.289), (DF3 [coconut] = 0.805), and (DF15 [quince] = 0.564). The findings indicate that the Pb, Cd, and Cr concentrations in the dry fruits of DF15 (quince) peaked followed by DF11 for apple and DF1 for peach, in that order. For the dry fruits, the lowest concentrations of Pb, Cd, and Cr were recorded in DF3, DF4, DF7, DF9, DF11, DF2, DF3, DF8, DF2, DF7, DF9, and DF11, respectively. According to these findings, certain dried fruits have extremely high levels of chromium contamination. Despite being within permissible bounds, lead poses a health risk. Cadmium and chromium surpassed the permitted limit in many samples, indicating the potential use of tainted water in manufacturing or agriculture contaminated by the environment or industry while being packaged or transported. These metals are poisonous, because they can damage the kidneys, liver, and nervous system when they accumulate in the body among other long‐term conditions. Results of EDI based on Pb, Cd, and Cr levels in 15 different dried fruit samples made in Iran and collected from Iraqi marketplaces are shown in Table 2. When dry fruit samples are used, the EDI averages for Pb, Cd, and Cr concentrations along with the standard error (S.E.) were 0.4584 mg/kg per day. Table 2 indicates that the daily doses were 0.1502 mg/kg and 0.2532 mg/kg, respectively. In addition, the average TQH for Pb, Cd, and Cr levels in dried fruit samples (Table 3) with S.E. was 0.131, 0.1364, and 0.084, respectively. Additionally, as shown in Table 3, the study’s HI results for dry fruits had an average of 0.3544 and varied from 0.056 to 0.755.

**Table 1:** Findings from studies on the concentrations of Pb, Cd, and Cr in various dry fruits in Iraqi markets.

No.	Sample name	Sample code	Concentrations in unit mg/kg		
			Pb	Cd	Cr
1	Peach	DF1	1.093	0.315	1.289
2	Fig	DF2	0.900	ND^∗^	ND^∗^
3	Coconut	DF3	ND^∗^	ND^∗^	0.805
4	Grape (raisins)	DF4	ND^∗^	0.331	0.242
5	Orange	DF5	0.450	ND^∗^	0.161
6	Sour Nomi	DF6	0.900	0.032	0.322
7	Pear	DF7	ND^∗^	0.378	ND^∗^
8	Watermelon	DF8	1.029	ND^∗^	0.242
9	Prune	DF9	ND^∗^	0.079	ND^∗^
10	Berry	DF10	0.750	0.173	0.081
11	Apple	DF11	ND^∗^	0.897	ND^∗^
12	Kiwi	DF12	1.051	0.378	0.403
13	Banana	DF13	0.600	0.189	0.483
14	Pomegranate	DF14	0.900	0.094	0.725
15	Quince	DF15	1.951	ND^∗^	0.564
Average ± S.E			0.6416 ± 0.80	0.1910 ± 0.43	0.3544 ± 0.59
Maximum permissible limits [29–31]			10	0.05	0.12

^∗^ND is not detected.

**Table 2:** The concentrations of Pb, Cd, and Cr in many dried fruits sold in Iraqi marketplaces cause EDI.

No.	Sample of code	EDI (mg/kg per day)		
		Pb	Cd	Cr
1	DF1	0.781	0.225	0.920
2	DF2	0.643	0.000	0.000
3	DF3	0.000	0.000	0.575
4	DF4	0.000	0.236	0.173
5	DF5	0.322	0.000	0.115
6	DF6	0.643	0.023	0.230
7	DF7	0.000	0.270	0.000
8	DF8	0.735	0.000	0.173
9	DF9	0.000	0.056	0.000
10	DF10	0.536	0.124	0.058
11	DF11	0.000	0.641	0.000
12	DF12	0.750	0.270	0.288
13	DF13	0.429	0.135	0.345
14	DF14	0.643	0.067	0.518
15	DF15	1.394	0.000	0.403
Average ± S.E		0.458 ± 0.67	0.150 ± 0.38	0.253 ± 0.50
Maximum permissible limits [3]		3.57	1.0	3.0

**Table 3:** The amounts of Pb, Cd, and Cr in several dry fruits sold in Iraqi markets led to THQ and HI findings.

No.	Sample code	THQ			HI
		Pb	Cd	Cr	
1	DF1	0.223	0.225	0.307	0.755
2	DF2	0.184	0.000	0.000	0.184
3	DF3	0.000	0.000	0.192	0.192
4	DF4	0.000	0.236	0.058	0.294
5	DF5	0.092	0.000	0.038	0.130
6	DF6	0.184	0.023	0.077	0.283
7	DF7	0.000	0.270	0.000	0.270
8	DF8	0.210	0.000	0.058	0.268
9	DF9	0.000	0.056	0.000	0.056
10	DF10	0.153	0.124	0.019	0.296
11	DF11	0.000	0.641	0.000	0.641
12	DF12	0.214	0.270	0.096	0.580
13	DF13	0.123	0.135	0.115	0.372
14	DF14	0.184	0.067	0.173	0.424
15	DF15	0.398	0.000	0.134	0.532
Average ± S.E		0.131 ± 0.36	0.136 ± 0.36	0.084 ± 0.28	0.351 ± 0.59
Maximum permissible limits [22]		1	1	1	1

Each dried fruit sample utilized in this study has its CR values listed in Table 4, as determined by equation (4). The mean values of CR × 10‐7 for Pb, Cd, and Cr were 42.64 ± 11.11, 0.016 ± 0.003, and 8.41 ± 2.68, respectively, as indicated by the data in Table 4, while the results of TCR × 10^−6^ ranged from 0.002 to 16.90, with an average of 5.106 ± 1.10.

**Table 4:** TCR findings for the concentrations of Pb, Cd, and Cr in various dry fruits offered for sale in Iraqi markets in relation to their cancer risk.

Sample code	CT × 10^−7^			TCR × 10^−6^
	Pb	Cd	Cr	
DF1	155.09	0.027	13.86	16.90
DF2	0.00	0.022	0.00	0.00
DF3	96.93	0.000	0.00	9.69
DF4	29.08	0.000	14.55	4.36
DF5	19.39	0.011	0.00	1.94
DF6	38.77	0.022	1.39	4.02
DF7	0.00	0.000	16.63	1.66
DF8	29.08	0.026	0.00	2.91
DF9	0.00	0.000	3.47	0.35
DF10	9.69	0.019	7.62	1.73
DF11	0.00	0.000	39.50	3.95
DF12	48.47	0.026	16.63	6.51
DF13	58.15	0.015	8.32	6.65
DF14	87.23	0.022	4.16	9.14
DF15	67.85	0.049	0.00	6.79
Average ± S.E	42.64 ± 11.11	0.016 ± 0.003	8.41 ± 2.68	5.106 ± 1.10
Range limit [32, 33]	10^−4^–10^−6^			

**Figure 5:**
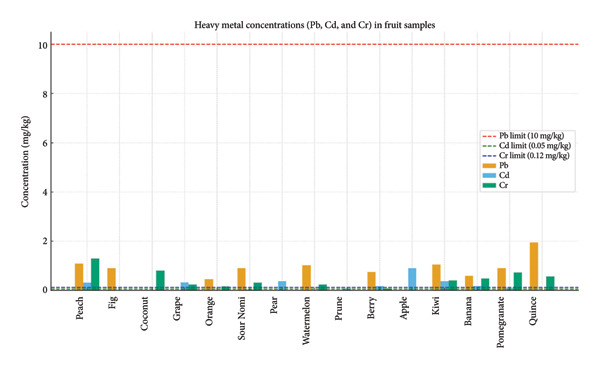
Fruit sample findings for heavy metals (Pb, Cd, and Cr) are compared to the uppermost allowable values.

Figure 5 shows the findings of the Pb, Cd, and Cr concentrations in the fruit samples used in this investigation in comparison with the maximum allowable limits for each metal, which are shown by dashed lines.

Table 1 lists the levels of heavy metals in different dried fruits that are sold in Iraqi markets. The greatest concentration of lead (Pb) is found in DF15 (quince), which has an average of 0.6416 mg/kg and a maximum of 10 mg/kg [29]. All of these values are within the acceptable range. However, lead’s presence in the majority of samples suggests a modest level of contamination that could become problematic over time. (CD) DF11 (apple) has the greatest concentration of 0.897 mg/kg, the average is 0.1910 mg/kg, and the maximum level is 0.05 mg/kg [30], which is far higher than what is allowed in a number of tests, such as DF1, DF11, and DF13, signifying extremely high levels of cadmium pollution. Significant chromium contamination was found in numerous samples, with the highest concentration of Cr in DF1 (peach) being 1.289 mg/kg, the average being 0.3544 mg/kg, and the maximum being 0.12 mg/kg [31], with glaring departures from the acceptable threshold.

**Figure 6:**
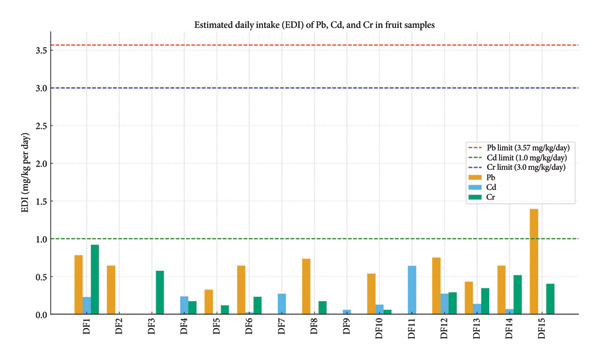
Comparing the EDI results for fruit samples with the maximum permissible limits.

Table 2 and Figure 6 display EDI results for Pb, Cd, and Cr in several dried fruits sold in Iraqi marketplaces. Figure 6 examines the effects of EDI brought on by Pb, Cd, and Cr concentrations in the study’s fruit samples with the maximum permissible limits for each metal, which are shown as dashed lines. The highest EDI values are obtained in DF15 (quince), DF1 (peach), and DF12 (kiwi), and the average values were Cd: 0.1502, Cr: 0.2532, and Pb: 0.4584 (mg/kg/day). Therefore, from Figure 6, all of the study’s samples fell inside the uppermost allowable bounds [3], and the values are 3 mg/kg daily for Cr, 1 mg/kg daily for Cd, and 3.57 mg/kg daily for Pb.

**Figure 7:**
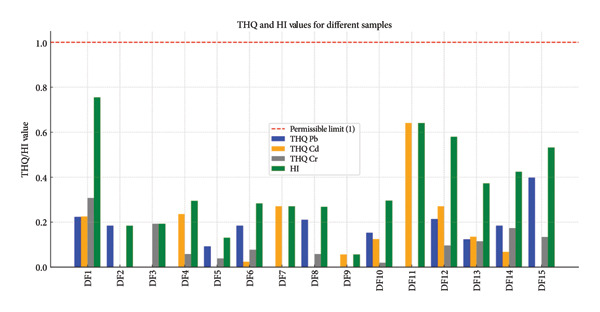
Fruit sample THQ and HI readings are compared to the uppermost allowable limits.

THQ and HI for Pb, Cd, and Cr results shown in Figure 7 and Table 3 are displayed in several dried fruits sold in Iraqi marketplaces. Figure 7 shows the comparison between the THQ and HI values in fruit samples from this investigation with the maximum allowable levels for each metal, which is one [22]. Additionally, the noncarcinogenic THQ was found to be at its maximum in DF11 = 0.641 (the closest point to risk); the average THQ was 0.131 for Pb, 0.1364 for Cd, and 0.084 for Cr, although the values were below 1. High values found in samples, such as DF11 and DF15, suggest a possible health risk associated with prolonged use. The HI ranges from 0.056 to 0.755, with an average of 0.351. While these values fall below the risk threshold (< 1), they do approach the red line in certain samples. A level of trace metals (Pb, Cd, and Cr) in imported drained fruits (peach, fig, coconut, grape, orange, Sour Nomi, pear, watermelon, prune, berry, apple, kiwi, banana, pomegranate, and quince) sold in the Iraqi market was measured using AAS. The results showed that samples of dried fruit, which are common in the study area, possess several elements in varying proportions. Nevertheless, the THQ results showed that the local populace was not at risk because the levels were less than one.

**Figure 8:**
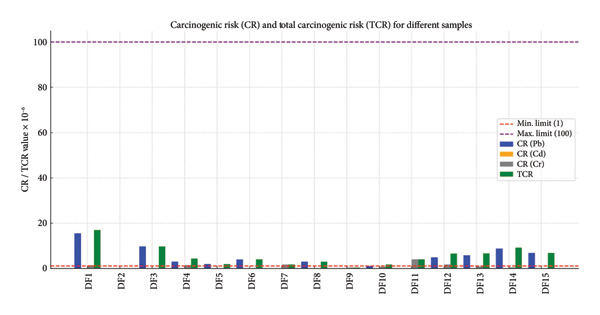
Comparison of the CR and TCR results in fruit samples with the maximum permissible limits.

A number of dried fruits offered for sale in Iraqi marketplaces exhibit CR and TCR as a result of Pb, Cd, and Cr, as shown in Table 4 and Figure 8. Figure 8 compares the CR and TCR results in fruit samples from this investigation with the 10^−6^ to 10^−4^ range of allowable limits for each metal [32, 33].

Cadmium (Cd), lead (Pb), and chromium (Cr) are examples of heavy metals that are hazardous environmental contaminants. Through food, drink, or the air, they can enter the human body and may lead to serious health effects when accumulated in vital tissues. One of the most dangerous outcomes is the increased risk of cancer, caused by either direct or indirect stimulation of genetic or metabolic processes in cells. Lead is known to have indirect carcinogenic effects by disrupting normal cellular processes and suppressing tumor‐suppressor genes; Pb acts as a mimic for essential elements, such as calcium, which causes disruption in neural and hormonal signaling, and may lead to changes in gene expression, particularly under chronic exposure conditions. While cadmium (Cd) contributed to increased TCR in fewer samples, such as DF11 and DF12, its effect is still significant, has a long biological half‐life, meaning it accumulates slowly but persistently in body tissues, particularly in the kidneys and bones, and disrupts natural antioxidant enzymes, such as glutathione, creating an oxidized cellular environment that favors genetic mutations and tumor formation. It also interferes with DNA repair mechanisms, increasing the probability of cancer development. Chromium (Cr^+6^) is most toxic and carcinogenic; the high TCR values in several samples, especially DF1, DF3, and DF14, indicate that chromium is the primary contributor to elevated cancer risk; Cr^+6^ has a high permeability through cell membranes, and once inside, it induces strong intracellular oxidation, leading to DNA damage. This damage creates a cellular environment conducive to the formation of cancer cells, particularly in the liver, lungs, and kidneys. Several samples, including DF1, DF3, DF14, and DF15, exceeded the acceptable cancer risk range defined by the USEPA (1 × 10^−6^ to 1 × 10^−4^), suggesting genuine health risks that require action such as treatment of water sources or soil contaminated with these metals.

The data in Table 5 compare amounts of Pb, Cd, and Cr heavy metals in samples of dried fruit sold in Iraqi markets with the findings of other research conducted in other nations, including Jordan, Turkey, Serbia, and Spain. Table 5 shows that the average Pb concentrations in raisins, Cd, and Cr in the samples from this investigation are lower than those from previous studies. Additionally, fig samples had an average Pb concentration that is higher than in Jordan, Serbia, and Spain and lower than in Turkey. Furthermore, the amounts of Cd and Cr in raisins are greater than any of the values found in Table 5.

**Table 5:** Comparison of Pb, Cd, and Cr in dried fruits from this research with earlier research.

Type of fruits	Country	Concentrations (mg/kg)			Reference
		Pb	Cd	Cr	
Fig	Jordan	0.29	0.02	—	[34]
	Turkey	2.5	0.15	—	[35]
	Serbia	0.41	—	—	[36]
	Spain	0.03	0.02	0.11	[37]

Raisins	Turkey	2.01	0.12	—	[35]
	Serbia	0.37	0.03	—	[36]
	Spain	0.03	0.003	0.07	[37]

Fig	Iraq	0.900	ND	ND	Present study
Raisins		ND	0.331	0.242	

## 4. Conclusions

The study’s findings demonstrated that high levels of chromium (Cr) and cadmium (Cd) were found in several dried fruit samples that were offered for sale in Iraqi markets. The World Health Organization (WHO) and the Food and Agriculture Organization (FAO) have set permissible limits. Levels of lead (Pb) were still within permissible bounds. The average chromium and cadmium concentrations were 0.3544 mg/kg (acceptable limit: 0.12 and 0.1910 mg/kg, respectively; permissible limit: 0.05) with the highest values recorded in apple samples (Cd = 0.897) and peach samples (Cr = 1.289) despite the fact that all samples’ overall HI was below 1. Although there is no direct noncarcinogenic health harm to the typical consumer, the overabundance of Cd and Cr concentrations raises questions over chronic long‐term exposure, particularly for vulnerable populations, such as expectant mothers and children. Based on these findings, the study authors recommend increasing the frequency of inspections of dried fruit imports entering Iraqi markets, enforcement of customs regulations and food safety (especially heavy metals), a mandatory condition for certified certificates of analysis from importers concerning origin countries, an effort to promote awareness programs to educate locals about risks associated with heavy metal exposure and cumulative doses, and expanding future studies to involve larger sample sizes as well as including other locally made dried fruits and also assessing cancer risk that accumulates over long‐term exposure. The levels of the harmful metals (Pb, Cd, and Cr) in the dried fruits that were being evaluated fell within the permissible ranges established by several health organizations. From a public health standpoint, the metal amounts found in this study were determined to be comparable to those found in prior studies. Further investigation into this subject is also recommended to look at the elemental concentrations in processed foods, fruits, cereals, and other culinary products.

## Ethics Statement

Neither human nor animal use is associated with the research that was conducted.

## Conflicts of Interest

The authors declare no conflicts of interest.

## Author Contributions

All authors acknowledge that they have equal rights to this paper.

## Funding

The authors state that no financing should be mentioned.

## Data Availability

The data that support the findings of this study are available from the corresponding author upon request.
